# Comparative *in vitro* activity of aztreonam–avibactam and aztreonam plus ceftazidime–avibactam against *Stenotrophomonas maltophilia* complex

**DOI:** 10.1128/aac.01456-25

**Published:** 2026-03-24

**Authors:** Ashlan J. Kunz Coyne, Rachel Gray, Pavani Gonnabathula, Elizabeth S. May, Pranita D. Tamma, Alex Do

**Affiliations:** 1 University of Kentucky College of Pharmacy 15511, Lexington, Kentucky, USA; 2 Department of Pediatrics, University of Pennsylvania School of Medicine 6572, Philadelphia, Pennsylvania, USA; Providence Portland Medical Center, Portland, Oregon, USA

**Keywords:** *Stenotrophomonas maltophilia *complex, aztreonam–avibactam, ceftazidime–avibactam, aztreonam, semi-mechanistic pharmacodynamic modeling

## Abstract

Members of the *Stenotrophomonas maltophilia* complex are intrinsically multidrug-resistant pathogens that disproportionately affect critically ill patients. Aztreonam–avibactam (ATM–AVI), FDA approved in 2025, combines aztreonam (ATM; stable to L1 β-lactamase) with avibactam (AVI; an L2 β-lactamase inhibitor). Aztreonam plus ceftazidime–avibactam (ATM–CZA) has been used as a surrogate for ATM–AVI, but direct comparisons between the two regimens are lacking. Twenty-three clinical *S. maltophilia* complex isolates underwent broth microdilution (BMD) testing for ATM, ceftazidime, ATM–AVI, and ATM–CZA, with gradient diffusion performed in parallel for ATM–AVI and ATM–CZA. Static 24-h time-kill assays at humanized steady-state Cmax concentrations evaluated bactericidal activity (≥3-log₁₀ reduction). Semi-mechanistic pharmacodynamic modeling characterized growth kill dynamics by resistance determinants (*blaL1*, *blaL2*, *smeABC*). ATM–CZA and ATM–AVI MIC_50/90_ values for BMD were 1/2 and 2/4 mg/L, respectively. Both regimens were bactericidal against most isolates, with a mean paired difference of 0.09 log₁₀ colony-forming units (CFU)/mL (*P* = 0.83). Isolate-level variation was evident: ATM–AVI sustained killing, whereas ATM–CZA permitted regrowth for MD17639, −4.86 vs 0.13 log₁₀ CFU/mL, *P* = 0.019; MD17061, −2.61 vs 0.64 log₁₀ CFU/mL, *P* < 0.001). Conversely, ATM–CZA achieved greater reductions in MD17662 (−3.84 vs −1.95; *P* = 0.026), MD17047 (−4.27 vs −2.64; *P* = 0.021), and UK4 (−3.47 vs −1.58; *P* = 0.017). Modeling predicted ATM–AVI benefit in *blaL2* and ATM–CZA benefit against *blaL1* or *smeABC* dominant isolates, and diminished activity of both when mechanisms coexisted. ATM–AVI and ATM–CZA show comparable *in vitro* activity against *S. maltophilia* complex. Isolate-level heterogeneity warrants further study of genotype–phenotype relationships.

## INTRODUCTION

*Stenotrophomonas maltophilia* complex are intrinsically multidrug-resistant pathogens that cause severe infections in immunocompromised and critically ill patients, with mortality often exceeding 50% (1–4). The most common syndromes are pneumonia and bloodstream infection, which carry the highest risk of death, particularly in patients with hematologic malignancies (2, 3). These infections remain difficult to treat because of both limited drug options and the diverse resistance mechanisms of *S. maltophilia* complex.

Resistance is driven primarily by two chromosomally encoded β-lactamases: the inducible L1 metallo-β-lactamase, which hydrolyzes most β-lactams but does not efficiently hydrolyze aztreonam, and the L2 serine β-lactamase, which confers cephalosporin resistance (5–7). Efflux pumps such as *smeABC* provide an additional barrier to antibiotic activity (8, 9). Historically, therapy has relied on trimethoprim-sulfamethoxazole, tetracyclines, and fluoroquinolones, but resistance, intolerance, and variable efficacy have increasingly undermined these agents (3, 4, 10). These limitations have renewed interest in β-lactam-based regimens that directly target the enzymatic drivers of resistance.

Aztreonam (ATM) is unique in its stability to L1, while avibactam (AVI) inhibits L2, making ATM–AVI a rational, mechanism-based regimen (11, 12). FDA approval in 2025 marked a long-awaited therapeutic advance, with early *in vitro* studies demonstrating restored susceptibility in ATM-resistant isolates (5, 13). Before its availability, the Infectious Diseases Society of America endorsed ATM plus ceftazidime–avibactam (ATM–CZA) as a surrogate approach because it provided AVI to inhibit L2 while relying on ATM to bypass L1 (14). However, the extent to which ceftazidime contributes additional antibacterial activity remains uncertain (14, 15).

An additional concern is whether concurrent exposure to two β-lactams (ATM and ceftazidime) could increase β-lactamase induction and potentially diminish activity. This concept parallels the well-characterized AmpC induction paradigm in *Enterobacter cloacae*, where certain β-lactams dysregulate the AmpD/AmpR axis and drive AmpC hyperproduction (16–19). Although β-lactam-induced upregulation of *blaL1* and *blaL2* has been described in *S. maltophilia*, the induction potency of individual β-lactams, including ceftazidime and ATM, is less well defined than in Enterobacterales. As a result, the degree to which induction influences therapeutic efficacy in *S. maltophilia* remains uncertain (20–23).

More specifically, β-lactamase induction in *S. maltophilia* is regulated through a complex network in which exposure to β-lactams increases the intracellular pool of cell-wall recycling intermediates (such as 1,6-anhydro-N-acetylmuramyl peptides), which bind to and activate the LysR-type regulator AmpR, thereby upregulating expression of the chromosomal *blaL1* and *blaL2* β-lactamase genes (24–27). Mutations or dysregulation within *ampD* or *mrcA* can result in constitutive hyperproduction of these β-lactamases, thereby enhancing hydrolytic activity and multidrug resistance (19, 23). This inducible response suggests that concurrent exposure to multiple β-lactams (e.g., ATM and ceftazidime) could potentiate β-lactamase expression and alter therapeutic efficacy.

Now that ATM–AVI is available for clinical use, an outstanding question is whether it offers advantages over the surrogate ATM–CZA regimen and whether ceftazidime contributes meaningful activity within this combination. To address this, we directly compared ATM–AVI with ATM–CZA using MIC testing and 24-h static time-kill assays, supported by semi-mechanistic pharmacodynamic modeling across a panel of clinical isolates.

## MATERIALS AND METHODS

### Bacterial isolates

Twenty-three clinical *S. maltophilia* complex isolates were collected from patients with culture-confirmed infections hospitalized at UK HealthCare (Lexington, KY, USA) or MD Anderson Cancer Center (Houston, TX, USA). Strains were maintained at −80°C in skim milk powder reconstituted with glycerol until use. Each isolate underwent whole-genome sequencing (Genewiz, Azenta, South Plainfield, NJ). Resistance gene identification, including detection of β-lactamases (*blaL1*, *blaL2*) and efflux determinants (*smeABC*), was performed using the Comprehensive Antibiotic Resistance Database (28, 29) and the 1928 Diagnostics analysis platform (Mölndal, Sweden). Whole-genome alignment and single-nucleotide polymorphism (SNP)-based phylogenetic analysis were also conducted in the 1928 Diagnostics platform. The alignment percentage (ALN) represents the proportion of each genome that aligns to the selected reference genomes during whole-genome alignment, providing a quantitative measure of relatedness and coverage across divergent lineages. Because the *S. maltophilia* complex contains highly divergent lineages, analyses were performed using two reference genomes (*Stenotrophomonas maltophilia* ASM2201473v1 and *Stenotrophomonas* sp. T8 ASM5185308v1) to improve alignment across distant clades. SNP distances were calculated from aligned regions after automated removal of clustered SNPs to limit the influence of recombination.

### Antibiotic susceptibility testing

The *S. maltophilia* complex isolates were cultured on tryptic soy agar (TSA) plates and incubated at 37°C for 24 h prior to susceptibility testing. Colony counts were performed on TSA plates (Hardy Diagnostics, Santa Maria, CA, USA). Mueller–Hinton broth (MHB; Millipore Sigma, Darmstadt, Germany) supplemented with calcium and magnesium at 25 and 12.5 mg/L, respectively (CAMHB), was used for all minimum inhibitory concentration (MIC) determinations.

MICs of study antibiotics were determined in triplicate by manual broth microdilution (BMD) using a 96-well plate format at standard inoculum, following Clinical and Laboratory Standards Institute (CLSI) M07 and M100 guidelines (30). Modal MIC values were derived from the three technical replicate wells. For combination testing, ATM–AVI was prepared with AVI fixed at 4 mg/L, and ATM–CZA with ceftazidime fixed at 8 mg/L and AVI fixed at 4 mg/L. *Escherichia coli* ATCC 25922 and *Klebsiella pneumoniae* ATCC 700603 served as quality control organisms. Antibiotics tested were research-grade analytical powders, including ATM (TCI Development Co., Ltd., Lot ZGKGH-TR), AVI (Sigma-Aldrich, Lot 0000332928), and ceftazidime (AmBeed, Lot A516848). Stock antibiotic solutions were prepared in sterile water, 0.22-µm filtered, aliquoted, and stored at −80°C until use.

Gradient diffusion testing was performed in parallel using bioMérieux ETEST strips (ATM–AVI strips: lot 1010310270; CZA strips: lot 1010965560; ATM strips: lot 1011254510). ATM–CZA synergy testing was conducted using the strip cross method, as previously described and optimized for clinical implementation (31). Mueller–Hinton agar plates (Remel, Lenexa, KS) were inoculated with the same 0.5 McFarland suspension used for BMD. The ATM strip was placed on the agar first. The CZA strip was then laid perpendicularly across the ATM strip at the 8 mg/L mark. Plates were incubated under the same conditions and for the same durations as those used for BMD.

### Static time-kill assays

Static concentration 24-h time-kill assays were conducted in duplicate on the same day to evaluate the pharmacodynamic activity of ATM in combination with AVI or CZA against the clinical *S. maltophilia* complex isolates at a targeted starting inoculum of 10⁷ colony-forming units (CFU)/mL. A direct suspension of three well-isolated colonies was taken from a pure overnight broth culture and suspended in 5 mL of CAMHB. The suspension was incubated at 37°C with agitation until mid-logarithmic phase. Suspensions were then adjusted to a 0.5 McFarland standard in sterile saline and diluted in CAMHB to achieve the target inoculum (~10⁷ CFU/mL), which was verified by colony counts.

Antibiotic concentrations reflected steady-state humanized Cmax values derived from published pharmacokinetic studies of standard clinical dosing regimens (11, 13, 14, 32–35). ATM, ceftazidime, and AVI were each tested at fixed static concentrations corresponding to 55, 75, and 12 mg/L, respectively. For each isolate, a drug-free growth control (CAMHB without antibiotics) was run in parallel and sampled at the same time points as the treated conditions. Growth controls were required to demonstrate sustained bacterial growth (≥1.0–2.0 log₁₀ CFU/mL increase from baseline at 24 h) to validate each experiment.

Samples were collected at 0, 4, 8, and 24 h, serially diluted 10-fold in sterile saline, and plated in duplicate using the Interscience easy Spiral Dilute plater (Interscience, Saint-Nom-la-Bretèche, France). Plates were incubated at 37°C for 24 h, and colonies were enumerated with the Scan1200 HD automatic colony counter (Interscience). The lower limit of quantification (LLQ) was 100 CFU/mL (2.0 log₁₀ CFU/mL), and the lower limit of detection (LOD) was 2 CFU/mL.

Bactericidal activity was defined as a ≥3.0-log₁₀ reduction in bacterial burden from baseline at 24 h. Regrowth was defined a priori as a ≥1.0-log₁₀ decrease at 8 h relative to baseline, followed by a ≥1.0-log₁₀ increase from 8 to 24 h. Bacterial counts (CFU/mL) are reported as mean ± standard deviation. Time-kill curves were generated by plotting log₁₀ CFU/mL vs time to visualize the killing activity of ATM–AVI and ATM–CZA regimens. Exploratory per-isolate comparisons of 24-h bacterial counts between regimens were assessed using two-sample Welch’s *t*-tests, with *P* values interpreted descriptively.

### Semi-mechanistic pharmacodynamic modeling

#### Model development and software

Previously reported semi-mechanistic pharmacodynamic (PD) models were applied to describe bacterial growth and antibiotic-mediated killing using static 24-h time-kill data for ATM, AVI, ATM–AVI, and ATM–CZA across 23 *S. maltophilia* complex clinical isolates (36, 37). MonolixSuite (Lixoft, version 2024R1) was used for structural model development, parameter estimation, and simulations.

#### Data structure

The data set was formatted in a longitudinal structure for Monolix with one record per observation. The following variables were included: ID, unique isolate identifier; TIME, sampling time (h); DV, observed bacterial density (log₁₀ CFU/mL), drug concentration, and REG_B0_obs, baseline inoculum (CFU/mL at time 0). Drug concentrations were fixed to experimentally defined values and treated as external regressors: REG_ATM, ATM concentration (mg/L); REG_AVI, AVI concentration (mg/L); REG_CEFTAZIDIME, ceftazidime concentration (mg/L), bypassing explicit pharmacokinetic modeling.

#### Structural pharmacodynamic model

Bacterial dynamics were modeled using a logistic growth function with antibiotic-mediated killing. Let *B*(*t*) denote bacterial density (CFU/mL) at time *t*:


$$\frac{dB}{dt}=k_{g}\cdot B\left(1-\frac{B}{B_{max}}\right)-K_{drug}\left(t\right)\cdot B$$

where *k_g_* is the bacterial growth rate constant, *B*_max_ the carrying capacity, and *K*_drug_*(t*) the drug-mediated killing term.

#### Drug effect functions

Antibiotic kill was described using a sigmoidal *E*_max_ function:


$$K_{drug}\left(t\right)=\frac{E_{max}C^{\gamma }}{(EC_{50}{)}^{\gamma }+C^{\gamma }}$$

where *E*_max_ is the maximal kill rate, *C* is the external concentration, *EC*_50_ is the concentration producing 50% of maximal effect, and *γ* is the Hill coefficient.

AVI potentiates the β-lactam activity of ATM and ceftazidime by modifying their effective *EC*_50_ through L2 β-lactamase inhibition.


$$EC_{50,ATM,eff}=\frac{EC_{50,ATM,res}}{1+\frac{\left[AVI\right]}{EC_{50,AVI}}}\;and$$

$$EC_{50,ceftazidime,eff}=\frac{EC_{50,ceftazidime,res}}{1+\frac{\left[AVI\right]}{EC_{50,AVI}}},$$

where *EC*_50,ATM,eff_ and *EC*_50,ceftazidime,eff_ represent AVI modified potency.

#### ATM–AVI model

For ATM–AVI, bacterial kill was modeled as


$$\frac{dB}{dt}=k_{g}B\left(1-\frac{B}{B_{max}}\right)-\left(\frac{E_{max,ATM}C_{ATM}^{\gamma }}{{\left(EC_{50,ATM,eff}\right)}^{\gamma }+\;C_{ATM}^{\gamma }}\right)B$$

#### ATM–CZA model

For the triple combination, ATM and ceftazidime kill effects were modeled separately, with AVI modifying *EC*_50_ for both drugs. The total kill rate was additive:


$$\frac{dB}{dt}=k_{g}B\left(1-\frac{B}{B_{max}}\right)-\left[\frac{E_{max,ATM}C_{ATM}^{\gamma }}{{\left(EC_{50,ATM,eff}\right)}^{\gamma }+\;C_{ATM}^{\gamma }}+\frac{E_{max,ceftazidime}C_{ceftazidime}^{\gamma }}{{\left(EC_{50,ceftazidime,eff}\right)}^{\gamma }+\;C_{ceftazidime}^{\gamma }}\right]B$$

#### Observation model

Observed counts were log-transformed:


$$Y=log_{10}\left(B\right)+\epsilon$$

Residual variability was modeled using a combined additive and proportional error structure, selected based on likelihood ratio testing.

#### Parameter estimation and variability

*B*_max_ (system carrying capacity) and the Hill coefficient (γ) were fixed to avoid overparameterization and ensure model stability. Bacterial growth rate constant (*k_g_*), maximum kill rate (*E*_max_), and potency (*EC*_50_) were estimated, as they directly reflect growth dynamics. Between-isolate variability (IIV) was modeled as log-normally distributed random effects. The precision of parameter estimates was evaluated using a non-parametric bootstrap (500 replicates).

#### Model evaluation

Model adequacy was assessed by goodness-of-fit plots (observed vs predicted values, residuals vs time), visual predictive checks (1,000 replicates) stratified by regimen, and biological plausibility of parameter estimates.

#### Simulation scenarios

To evaluate how *S. maltophilia* resistance mechanisms impact pharmacodynamic outcomes, the final pharmacodynamic model was extended to simulate β-lactamase- and efflux-mediated resistance. Biological resistance was modeled by dynamically scaling the antibiotic potency parameter (*EC*_50_) using the resistance factor (β), which quantifies the fold change in drug efficacy associated with specific determinants. The approach of modifying *EC*_50_ to reflect resistance mechanisms builds on previous semi-mechanistic PK/PD modeling frameworks (11, 38–40). Each β-factor represents a hypothetical fold change in antibiotic potency associated with the presence of a given resistance determinant (e.g., *blaL1*, *blaL2*, or *smeABC*). β-factors were initialized as biologically plausible starting values but were ultimately estimated from the experimental time-kill data, allowing the model to quantify gene-specific impacts on susceptibility from the observed bacterial responses, which enables exploration of individual or combined resistance mechanisms without requiring direct experimental measurement of enzyme activity or efflux pump expression.

Each isolate was characterized using binary regressors indicating gene presence: REG_L1 = 1 for the L1 metallo-β-lactamase, REG_L2 = 1 for the L2 serine β-lactamase, and REG_SmeABC = 1 for *smeABC* presence. The L1 β-lactamase does not hydrolyze ATM directly, but its co-presence with the L2 β-lactamase can enhance total β-lactamase output. L2 directly hydrolyzes ATM and ceftazidime, and its inhibitory effect is reversed by AVI. The smeABC efflux system reduces intracellular antibiotic concentrations, further diminishing drug potency.

The general *EC*_50_ scaling relationship was defined as


$$EC_{50,ATM,res}=EC_{50,ATM}\times \left(1+\sum_{i}REG_{i}\cdot \beta_{i}\right)$$

$$EC_{50,ceftazidime,res}=EC_{50,ceftazidime}\times \left(1+\sum_{i}REG_{i}\cdot \beta_{i}\right)$$

where *REGᵢ* represents gene presence (1 = expressed, 0 = absent), and *βᵢ* is a multiplicative resistance factor, representing the fold change in antibiotic potency caused by the presence of specific genes.

Avibactam potentiation was incorporated for the effective *EC*_50_ in proportion to its concentration. These adjusted *EC*_50_ values were integrated into a sigmoidal *E*_max_ kill model coupled with logistic bacterial growth dynamics to simulate bacterial response under various resistance profiles. Simulations were performed for baseline, single, and combined mechanisms (L1, L2, SmeABC, L1+L2, L1+SmeABC, L2+SmeABC, and L1+L2+SmeABC). For each scenario, 1,000 virtual isolates were simulated under both regimens (ATM–AVI and ATM–CZA), and mean ± variability in bacterial density (log₁₀ CFU/mL) over 24 h were summarized.

## RESULTS

### Whole-genome analysis

**Fig 1:**
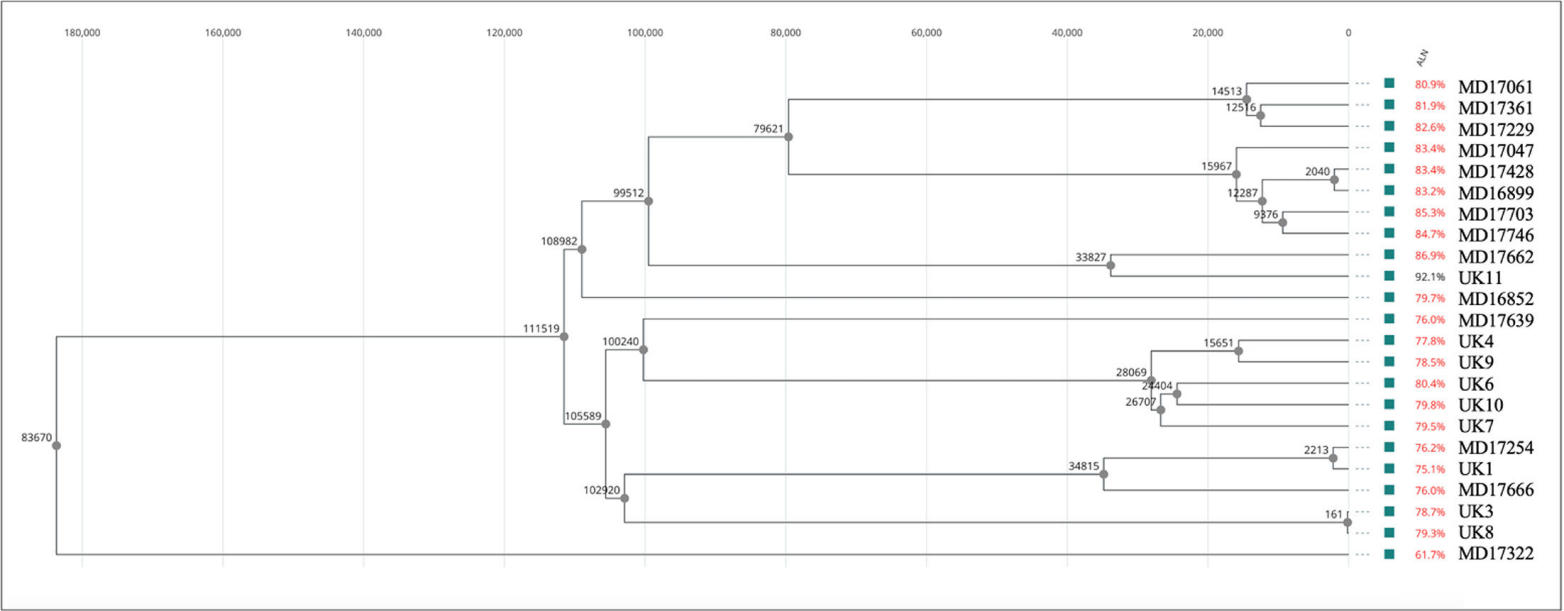
Phylogenetic tree based on whole-genome alignment of *Stenotrophomonas* isolates. Isolates clustered within the *S. maltophilia* complex, including *S. maltophilia*, *S. pavanii*, and *S*. sp. PAMC25021. ALNs are shown to the right of the tree.

Whole-genome alignment demonstrated substantial genomic diversity among the 23 *S. maltophilia* isolates, with ALNs ranging from approximately 62% to more than 90% (Fig. 1). Isolates with lower ALN values formed long and deeply separated branches, consistent with marked divergence from the reference sequences. Use of two reference genomes (ASM2201473v1 and ASM5185308v1) improved alignment coverage across distant lineages and allowed resolution of several distinct clades within the *S. maltophilia* complex. The isolates included in MIC and time-kill experiments were distributed throughout the phylogeny and did not cluster by clinical center, indicating that the experimental subset reflected the overall genomic diversity of the panel. Overall, the phylogeny confirms that the isolate collection spans multiple lineages within the *S. maltophilia* complex, including *S. maltophilia*, *Stenotrophomonas pavanii*, and *S.* sp. PAMC25021.

### Antimicrobial susceptibility

BMD MICs for ATM–CZA and ATM–AVI were determined for all 23 *S. maltophilia* complex isolates (Table 1). ATM–CZA MICs ranged from ≤0.25 to 8 mg/L, with MIC_50/90_ values of 1/2 mg/L. ATM–AVI MICs ranged from 1 to 64 mg/L, with MIC_50/90_ values of 2/4 mg/L. Gradient diffusion results showed close agreement with BMD for both combinations, generally within one doubling dilution of BMD values. Ceftazidime MICs ranged from 2 to >64 mg/L, while the MIC for aztreonam alone exceeded 64 mg/L for all isolates.

**TABLE 1:** *S. maltophilia* complex phenotypic and genotypic characteristics

Isolate number	Species	MIC (mg/L)^*a*^					Antibiotic resistance genes^*c*^		
		ATM–CZA		ATM–AVI		Ceftazidime^*b*^	*β*-lactamase		Efflux pump
							*bla*_L1_	*bla*_L2_	*smeABC*
		BMD	Gradient	BMD	Gradient	BMD			
MD16852	*S. maltophilia*	≤0.25	0.5	2	2	>64			
MD16899	*S. maltophilia*	≤0.25	0.25	2	2	>64			
MD17047	*S. pavanii*	≤0.25	0.5	2	2	>64			
MD17061	*S. pavanii*	2	2	2	2	>64			
MD17203	*S*. PAMC25021	≤0.25	0.25	1	1	2			
MD17229	*S. pavanii*	≤0.25	0.25	2	2	16			
MD17254	*S. maltophilia*	4	4	4	4	64			
MD17322	*S. maltophilia*	≤0.25	0.25	2	2	4			
MD17361	*S. pavanii*	1	1	2	2	4			
MD17428	*S*. PAMC25021	0.5	0.5	4	4	4			
MD17639	*S. maltophilia*	2	2	2	2	>64			
MD17662	*S. maltophilia*	2	2	4	4	>64			
MD17666	*S. maltophilia*	2	2	2	2	4			
MD17746	*S*. PAMC25021	≤0.25	0.5	2	2	8			
UK1	*S. maltophilia*	8	8	≥64	>256	>64			
UK3	*S. maltophilia*	2	2	4	4	64			
UK4	*S. maltophilia*	2	2	2	2	2			
UK6	*S. maltophilia*	2	2	2	2	8			
UK7	*S. maltophilia*	1	1	2	2	4			
UK8	*S. maltophilia*	0.5	0.5	1	1	8			
UK9	*S. maltophilia*	2	2	2	2	32			
UK10	*S. maltophilia*	≤0.25	0.25	2	2	4			
UK11	*S. maltophilia*	≤0.25	0.5	4	4	64			

^*a*^ Minimum inhibitory concentrations (MICs, mg/L) for aztreonam (ATM), as measured in combination with ceftazidime–avibactam (ATM–CZA) and ATM with avibactam (ATM–AVI), were determined in triplicate by BMD following CLSI guidelines, using a standardized inoculum of 10⁶ CFU/mL. For combination testing, the concentrations of the partner agents were held constant at 8 mg/L ceftazidime and 4 mg/L avibactam for both ATM–CZA and ATM–AVI assays. MIC values represent the mode of three replicates. Gradient strip assays were performed in parallel with BMD for both ATM–CZA and ATM–AVI.

^*b*^ Standalone BMD MICs for aztreonam and ceftazidime were also determined for all isolates. The MIC for ATM alone was >64 mg/L for every isolate tested and thus, not reported.

^*c*^ Antibiotic resistance genes (*blaL1*, *blaL2*, and *smeABC*) were identified via whole-genome sequencing of 23 *S. maltophilia *complex clinical isolates from UK HealthCare and MD Anderson Cancer Center. In the resistance-gene columns, gray indicates presence of *blaL1*, *blaL2*, and/or *smeABC*; blank cells denote absence of the corresponding wild-type gene.

### Antibiotic resistance genes

Twenty-two of twenty-three isolates carried at least one wild-type chromosomal β-lactamase. Most isolates possessed both *blaL1* and *blaL2* in their wild-type forms, while two isolates carried only a single wild-type enzyme (*blaL1* in MD16852; *blaL2* in MD17322). One isolate (UK1) lacked both wild-type β-lactamases. The wild-type smeABC efflux operon was present in 20 of 23 isolates, with UK1, MD16852, and MD17322 lacking this operon (Table 1).

### Static time-kill assays

**Fig 2:**
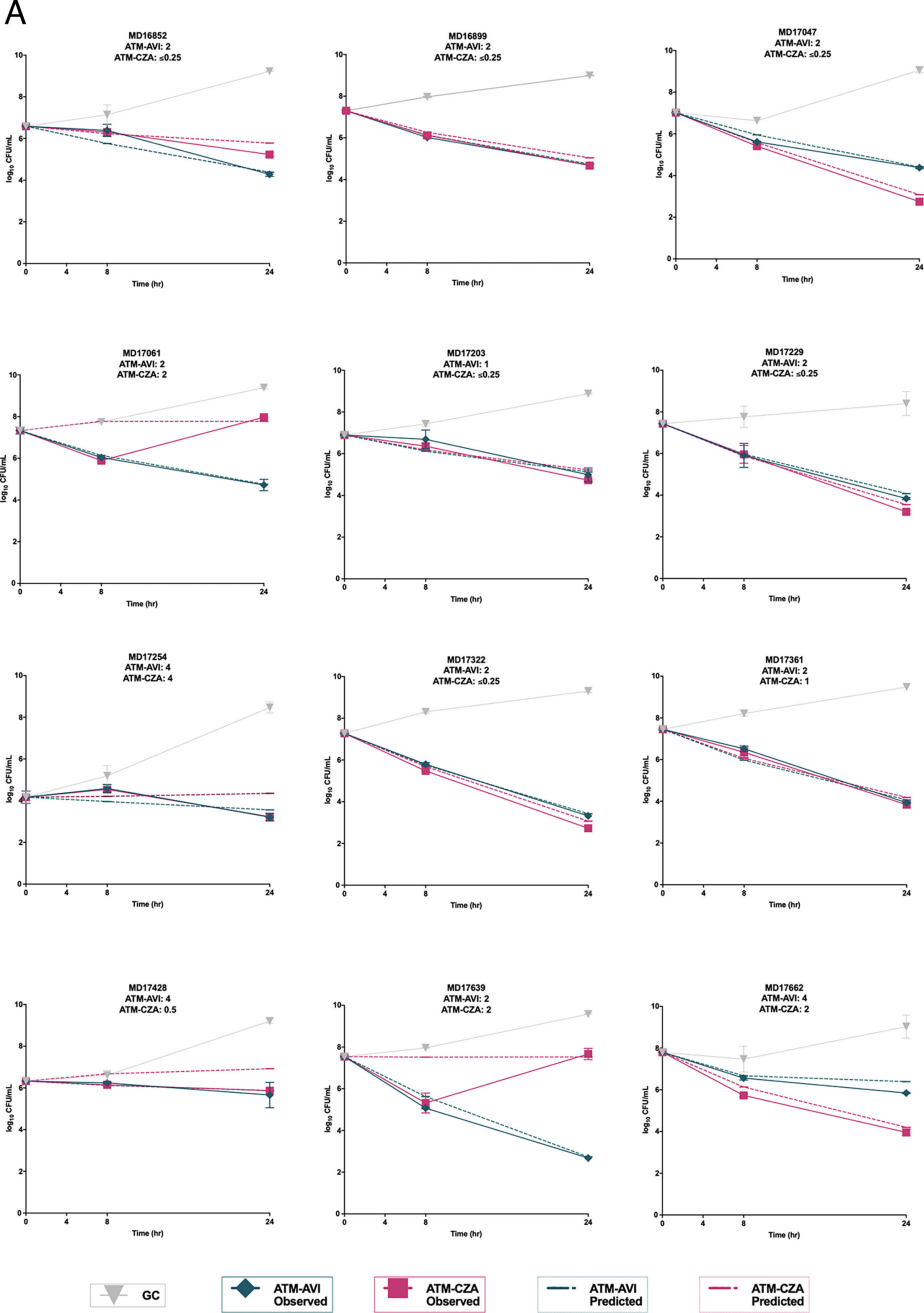

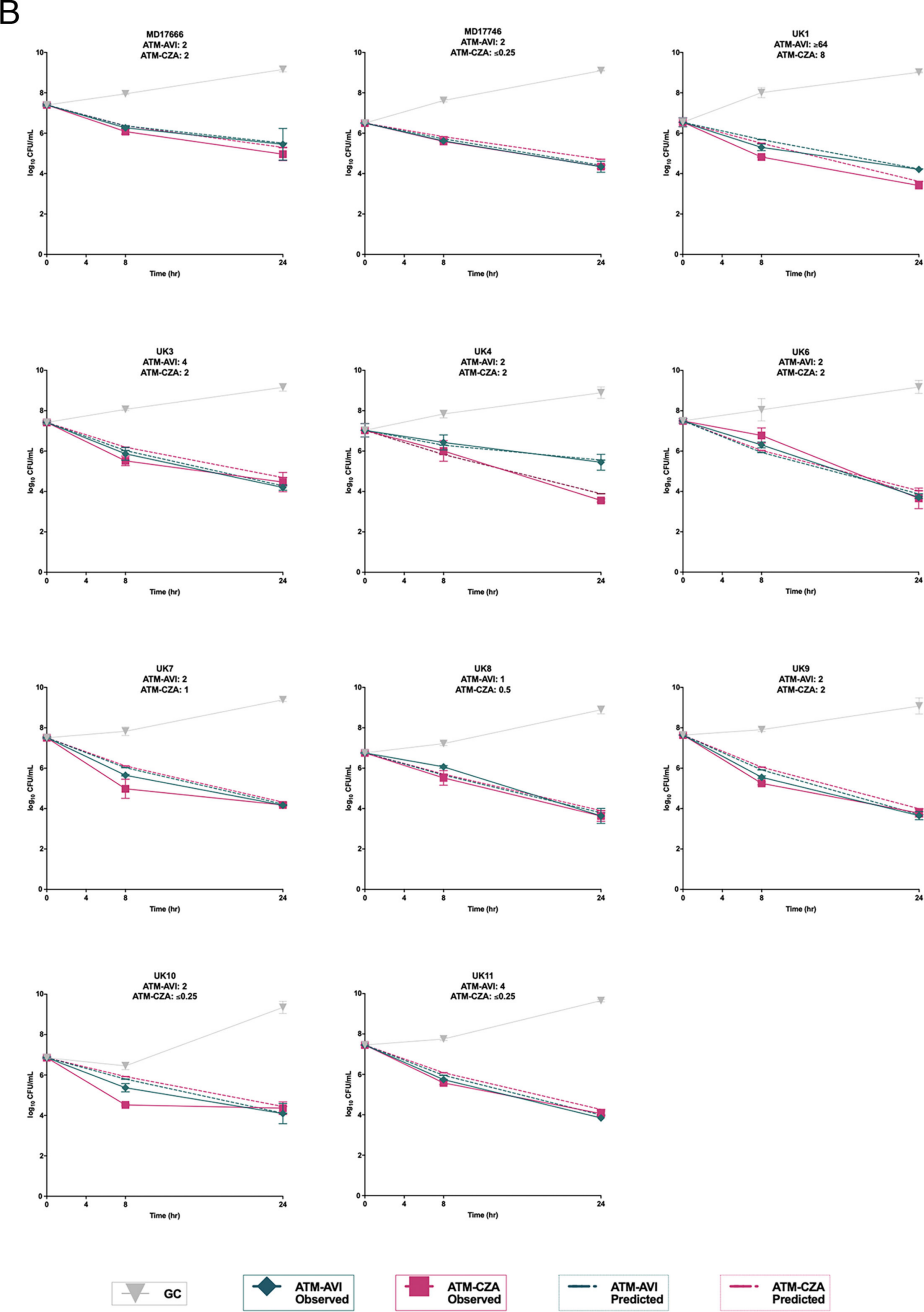
Observed and predicted time-kill curves from semi-mechanistic modeling comparing aztreonam–avibactam and aztreonam plus ceftazidime–avibactam against diverse *S. maltophilia* complex isolates over 24 h. (**A**) shows the first 12 isolates, and (**B**) shows the remaining 11 isolates. Time-kill curves represent mean bacterial counts (log₁₀ CFU/mL) from duplicate 24-h assays at a starting inoculum of 10⁷ CFU/mL, using humanized Cmax concentrations of ATM–AVI and ATM–CZA. Observed data (solid lines) are plotted with an LOD of 2 CFU/mL and an LLQ of 100 CFU/mL; predicted curves (dashed lines) are derived from semi-mechanistic pharmacodynamic modeling in MonolixSuite, accounting for L1, L2, and smeABC resistance mechanisms. Error bars indicate standard deviation where applicable. ATM–AVI, aztreonam–avibactam; ATM–CZA, aztreonam plus ceftazidime–avibactam.

Both regimens reduced bacterial burden relative to baseline in all 23 isolates at 24 h (Fig. 2), although two isolates demonstrated regrowth between 8 and 24 h with ATM–CZA. Cohort-level analysis showed a median paired difference (ATM–AVI—ATM–CZA) of 0.01 log₁₀ CFU/mL (IQR –0.21 to 0.54). The mean paired difference was –0.09 log₁₀ CFU/mL (95% CI –0.72 to 0.55; paired *t*-test, *P* = 0.83). ATM–AVI produced lower 24-h counts in 11 of 23 isolates (48%) and ATM–CZA in 12 of 23 (52%). Median 24-h counts were 4.20 log₁₀ CFU/mL with ATM–AVI and 4.06 log₁₀ CFU/mL with ATM–CZA, with corresponding median reductions of 2.64 and 3.13 log₁₀, respectively.

ATM–AVI achieved bactericidal activity in 10 of 23 isolates (43%) and was static in 13 of 23 (57%). No regrowth events occurred with ATM–AVI. ATM–CZA was bactericidal in 12 of 23 isolates (52%), static in 9 of 23 (39%), and exhibited regrowth in 2 of 23 (9%) (MD17061 and MD17639).

The largest regimen-specific differences favored ATM–AVI in MD17639 (Δ = –4.99 log₁₀) and MD17061 (Δ = –3.25). ATM–CZA was favored for UK4 (Δ = +1.89), MD17662 (Δ = +1.89), and MD17047 (Δ = +1.63). Exploratory per-isolate Welch’s *t*-tests identified six isolates with *P* < 0.05: lower 24-h counts with ATM–AVI in MD16852, MD17061, and MD17639, and lower counts with ATM–CZA in MD17047, MD17229, and MD17662.

### Semi-mechanistic model prediction

Semi-mechanistic modeling in MonolixSuite reproduced time-kill profiles with deviations <1 log₁₀ CFU/mL, which is within expected experimental variability for static time-kill assays, and captured regrowth events in MD17061 and MD17639 with ATM–CZA (Fig. 2). Model parameters, including *E*_max_ (maximum kill rate) and *EC*_50_ (concentration for 50% of *E*_max_), were optimized for each regimen and genetic background. *Emax* was generally higher for ATM–CZA in the no-gene scenario and in *smeABC*-dominant isolates, indicating faster kill. Lower *E*_max_ values were observed for ATM–CZA in *blaL1*-, *blaL2*-, and combined-gene scenarios, and *EC*_50_ values were lower for ATM–AVI in *blaL2*-only isolates.

Scenario analyses demonstrated consistent overarching patterns. Across backgrounds lacking major resistance determinants, both regimens were effective, with ATM–AVI producing slightly lower observed and predicted 24-h counts (approximately 0.5 to 1 log₁₀). In *blaL1*-only or *smeABC*-only backgrounds, ATM–CZA generally produced lower counts (typically 0.5 to 3 log₁₀). In *blaL2*-only backgrounds, both regimens retained activity, and ATM–AVI consistently produced lower counts (1 to 2 log₁₀). When multiple mechanisms coexisted, including *blaL1* plus *blaL2*, *smeABC* combinations, or triple profiles, both regimens showed reduced activity, and relative differences were isolate-dependent. ATM–CZA produced lower counts in several combined-mechanism backgrounds, and ATM–AVI produced lower counts in *smeABC*-negative isolates such as UK1.

## DISCUSSION

This study provides the first direct comparison of ATM–AVI and ATM–CZA against a diverse panel of *S. maltophilia* complex clinical isolates. By integrating MIC testing, static 24-h time-kill assays, and semi-mechanistic pharmacodynamic modeling, we evaluated how these regimens perform across wild-type backgrounds of *blaL1*, *blaL2*, *smeABC*, and combinations thereof. Collectively, the data suggest that ATM–AVI and ATM–CZA exhibit comparable overall *in vitro* activity in tested static concentration assays, with regimen-specific differences emerging at the individual isolate and genotype levels.

### MIC findings and mechanistic considerations

The MIC results demonstrated overlapping activity between the two regimens. ATM–CZA MIC values were modestly lower than those for ATM–AVI, which aligns with prior studies evaluating ATM, AVI, and ceftazidime-containing combinations (15, 41). Several mechanistic features of *S. maltophilia* may have contributed to these differences. Ceftazidime is a preferred substrate of the L1 metallo-β-lactamase, and most molecules are hydrolyzed efficiently (1, 42–44). Even so, a fraction of ceftazidime may evade L1 long enough to reach PBPs, and with L2 inhibited by AVI, those molecules would retain intrinsic activity (15, 41).

In the ATM–CZA combination, this residual ceftazidime activity would accompany the activity of ATM, whereas ATM–AVI relies solely on ATM. Although the extent of ceftazidime’s contribution is not fully defined, the presence of an additional β-lactam with partial activity provides a plausible explanation for the modestly lower MICs observed with ATM–CZA relative to ATM–AVI across diverse genetic backgrounds in our study (1, 42, 44, 45). Together, these interactions provide a mechanistic context for the modest MIC differences observed between regimens.

In a study of 68 ATM-nonsusceptible isolates, AVI restored susceptibility to ATM in nearly 90% of isolates and reduced the ATM–AVI MIC₅₀ from 64 to 2 mg/L (15). Additional diagnostic studies have shown that ATM–CZA often yields lower MIC values than ATM–AVI using gradient diffusion and superposition methods (41). Our findings confirm this pattern across a genetically diverse isolate set. However, lower MIC values for ATM–CZA did not consistently translate to superior suppression in dynamic assays. Several isolates with low ATM–CZA MICs demonstrated regrowth during 24-h exposure, whereas ATM–AVI prevented rebound. These observations reinforce that MIC values may not fully capture intracellular pharmacodynamics in organisms that upregulate β-lactamases, accumulate inhibitors, or adjust efflux output in a concentration-dependent manner (45, 46).

### Time-kill activity

Across the 23-isolate cohort, the two combinations displayed similar 24-h inhibitory and bactericidal profiles, with mean and median paired differences approaching zero. Both regimens achieved bactericidal activity in a subset of isolates, and neither regimen was uniformly superior. However, isolate-level heterogeneity was pronounced. ATM–AVI prevented regrowth in isolates where ATM–CZA permitted rebound (MD17061 and MD17639), whereas ATM–CZA achieved greater reductions in others (UK4, MD17662, MD17047). These isolate-specific behaviors demonstrate that MIC_50_ metrics or median kill values do not represent the full range of possible responses within the *S. maltophilia* complex and emphasize the importance of dynamic experimental systems.

### Mechanisms underlying time-kill activity-MIC discordance

Several mechanistic factors likely contributed to the observed discrepancies between MIC results and 24-h killing. Efflux kinetics may differ under dynamic exposure; in isolates carrying *smeABC* for which ceftazidime is a substrate but not aztreonam, ceftazidime may be exported more rapidly than ATM, reducing the extent of L1 saturation during time-dependent killing, even if MIC values appear favorable (47). Additionally, inducible production of L1, L2, or SmeABC over time during prolonged antibiotic exposure may alter intracellular drug availability in ways not reflected by static MICs. These mechanisms may explain why some isolates with modest ATM–AVI MICs responded more favorably to ATM–AVI in time-kills and why some isolates with low ATM–CZA MICs demonstrated regrowth despite apparently favorable susceptibility profiles.

### Semi-mechanistic modeling

Semi-mechanistic pharmacodynamic modeling reproduced observed time-kill trajectories within less than 1 log₁₀ CFU/mL and captured the regrowth seen with ATM–CZA in isolates MD17061 and MD17639. The model allowed estimation of regimen-specific *E*_max_ and *EC*_50_ values across defined genetic backgrounds and supported the experimental patterns identified in static assays. In *blaL2* dominant backgrounds, ATM–AVI consistently produced lower predicted 24-h counts and lower *EC*_50_ values, reflecting greater potency when L2 hydrolysis is neutralized by AVI. In *blaL1* dominant or *smeABC* dominant scenarios, ATM–CZA produced lower predicted bacterial densities and exhibited higher *E*_max_ values, consistent with more efficient maximal killing in settings where ceftazidime can occupy L1 or interact with efflux pathways. When multiple mechanisms coexisted, including combinations of *blaL1*, *blaL2*, and *smeABC*, both regimens demonstrated reduced activity, and predicted differences were isolate dependent. These genotype-linked shifts in *E*_max_ and *EC*_50_ suggest that L1 primarily influences maximal kill, L2 primarily influences potency, and SmeABC decreases intracellular antibiotic exposure, which together shape the distinct kill curve profiles observed for each regimen. Overall, the modeling framework corroborated the experimental findings by showing that differential interactions with L1, L2, and SmeABC drive regimen-specific activity patterns and that neither combination has a uniform advantage across the *S. maltophilia* complex.

### Limitations and clinical relevance

Multiple limitations warrant consideration. First, static time-kill assays maintain constant antibiotic concentrations and a 24-h exposure horizon, which do not reflect clinical pharmacokinetics or multi-day treatment. Second, the target inoculum (10^7^ CFU/mL) reflects high-burden infection but may not generalize to all infection types. Although the isolate panel was genetically diverse, it did not encompass global diversity. Third, gene expression was not measured directly. Additionally, dynamic models that simulate human concentration–time profiles were not conducted, and it will be important to expand upon our findings. Finally, the Cmax-level exposures used do not reproduce the concentration–time fluctuations that occur clinically or the prolonged infusions commonly used to optimize beta-lactam activity. Although both combinations achieve peak plasma concentrations that exceed the modal MICs observed in this study, their short half-lives limit time above the MIC *in vivo*.

At present, clinical laboratories do not routinely detect *blaL1*, *blaL2*, or *smeABC*, limiting the ability to tailor therapy based on genotype. Clinical studies will be important to determine whether the isolate-specific differences identified here translate into differences in clinical response. Improved diagnostic tools capable of detecting these intrinsic resistance determinants may help refine regimen selection in the future.

### Future directions and summary

Future studies should incorporate humanized, multi-day dynamic models to evaluate the durability of suppression, emergence of resistance, and optimal dosing strategies for ATM–AVI and ATM–CZA. Clinical studies stratified by resistance genotype will help determine whether genotype can inform regimen selection. In summary, ATM–AVI and ATM–CZA demonstrated comparable overall activity against *S. maltophilia* complex, with important isolate- and genotype-dependent variation. ATM–AVI tended to perform better in *blaL2*-dominant contexts, whereas ATM–CZA tended to perform better in *blaL1*- and efflux-dominant settings. These findings underscore the value of combining MIC, dynamic PK/PD, and mechanistic modeling approaches and highlight the need for further clinical investigation.
